# Expression, Purification, Characterization and Cellular Uptake of MeCP2 Variants

**DOI:** 10.1007/s10930-022-10054-9

**Published:** 2022-05-12

**Authors:** Alexander V. Beribisky, Hannes Steinkellner, Sofia Geislberger, Anna Huber, Victoria Sarne, John Christodoulou, Franco Laccone

**Affiliations:** 1 Institute of Medical Genetics, Center for Pathobiochemistry and Genetics, Medical University of Vienna, Währinger Straße 10, A-1090 Vienna, Austria; 2 Department of Paediatrics, Murdoch Children’s Research Institute, Royal Children’s Hospital, University of Melbourne, Melbourne, Australia; 3 Vienna Doctoral School of Pharmaceutical, Nutritional and Sport Sciences (PhaNuSpo), University of Vienna, Althanstraße 14, A-1090 Vienna, Australia

**Keywords:** MeCP2, Rett Syndrome, TAT-fusion proteins, Cell penetrating peptide, Protein structural characterization

## Abstract

The transcriptional regulator Methyl-CpG-binding protein 2 (MeCP2) is an intrinsically disordered protein, mutations in which, are implicated in the onset of Rett Syndrome, a severe and debilitating neurodevelopmental disorder. Delivery of this protein fused to the cell-penetrating peptide TAT could allow for the intracellular replenishment of functional MeCP2 and hence potentially serve as a prospective Rett Syndrome therapy. This work outlines the expression, purification and characterization of various TAT-MeCP2 constructs as well as their full-length and shortened eGFP fusion variants. The latter two constructs were used for intracellular uptake studies with subsequent analysis via western blotting and live-cell imaging. All purified MeCP2 samples exhibited high degree of stability and very little aggregation propensity. Full length and minimal TAT-MeCP2-eGFP were found to efficiently transduce into human dermal and murine fibroblasts and localize to cell nuclei. These findings clearly support the utility of MeCP2-based protein replacement therapy as a potential Rett Syndrome treatment option.

**Supplementary Information:**

The online version contains supplementary material available at 10.1007/s10930-022-10054-9.

## Introduction

Rett Syndrome (RTT) is a neurodevelopmental disorder affecting 1 in 10,000 live female births [1]. The disease manifests itself within six to eighteen months after birth and is characterized by deterioration of cognitive, communication and motor skills. This, in turn, results in a loss of purposeful upper limb use, replaced by stereotypic motions, and gait abnormalities. Other symptoms include the emergence of autistic-like features, abnormal muscle tone, seizures as well as breathing disturbances when in a waking state [2].

**Fig. 1:**
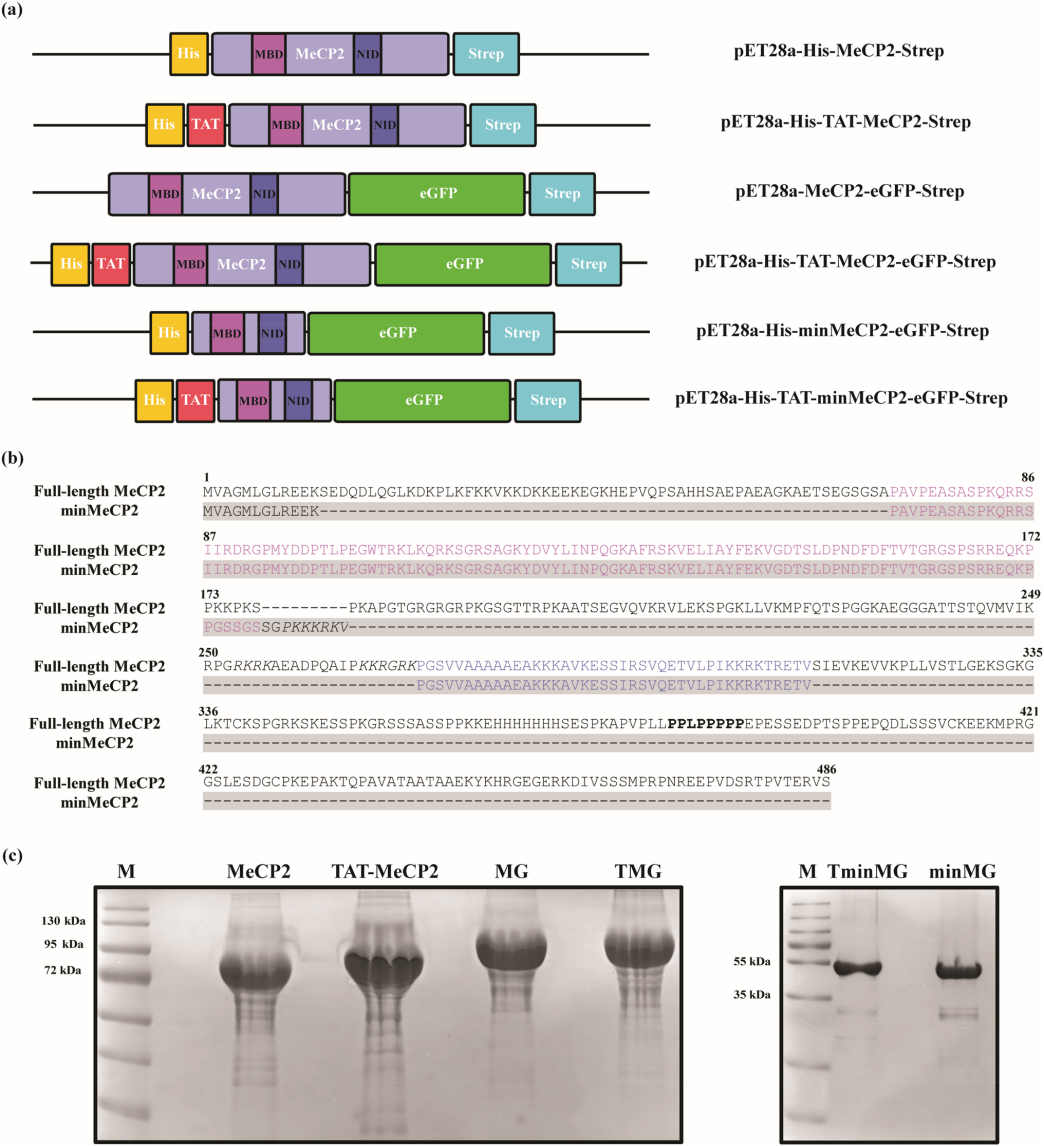
**a** Schematic representation of MeCP2, TAT-MeCP2, MeCP2-eGFP, TAT-MeCP2-eGFP, minMeCP2-eGFP and TAT-minMeCP2-eGFP constructs. **b** Sequence alignment of the full-length (no highlighting) and minimal MeCP2 (highlighted in grey). Sequences corresponding to the MBD and NID are colored in light and dark purple respectively. Nuclear localization signals are italicized. A polyproline stretch in the full-length MeCP2 sequence is marked in bold. **c** SDS-PAGE of MeCP2 constructs. M–Marker, MeCP2, TAT-MeCP2, MG–MeCP2-eGFP, TMG–TAT-MeCP2-eGFP; M–Marker, TminMG–TAT-minMeCP2-eGFP, minMG–minMeCP2-eGFP

RTT onset has been linked to loss-of function mutations in the *MECP2* gene which is located on the X-chromosome [1]. Notably, multiple copies of this gene, lead to the onset of a distinct neurological disorder termed *MECP2* duplication syndrome [3]. The *MECP2* gene encodes for Methyl-CpG-binding protein 2 (MeCP2), a multifunctional intrinsically disordered protein (IDP) [1]. While being present in various tissue types, MeCP2 plays an integral role in the central nervous system (CNS). MeCP2 is known to act as a regulator of gene expression by increasing and silencing transcription [4] through interactions with various methylated and unmethylated DNA sequences, and by modulating chromatin remodeling. These functions have largely been attributed to two structured portions of MeCP2. One such part is the methyl binding domain (MBD) while the other is the transcriptional repression domain (TRD). The former has been implicated in the aforementioned protein-DNA contacts [5], while the latter was demonstrated to be involved in protein–protein interactions, such as SIN3A [6] and TBLR1 [7] as well as with other elements involved in the repression of gene expression [8, 9]. These MeCP2 functions were shown to be crucial in the context of RTT onset. In fact, it has been suggested that a truncated MeCP2 construct (Fig. 1b) containing the MBD, as well as a portion of the TRD termed the NCoR/SMRT Interaction Domain (NID) [10], was found to rescue RTT phenotypes in mice [11].

It has already been shown that RTT may be a reversible condition. Experiments performed in RTT mouse models, suggest that restoring functional MeCP2 presence in the cells of the CNS can lead to the amelioration of numerous RTT symptoms [12]. Potential methods to achieve this goal include, but are not limited to: Gene correction, virus-mediated *MECP2* delivery, and MeCP2 expression from the inactivated X-chromosome [3]. More recently, clustered regularly interspaced short palindromic repeats (CRISPR) technology was used to attempt to correct the disease relevant regions in the *MECP2* sequence [13, 14].

Another potential treatment option is protein replacement therapy. It involves delivering MeCP2 into the affected cells with this protein being tethered to a cell penetrating peptide (CPP). One such CPP is TAT—an eleven amino-acid residue motif derived from the Tat protein in the human immunodeficiency virus 1 (HIV-1). TAT-fusion proteins have been shown to cross the blood–brain barrier (BBB) and consequentially might possess the potential to treat CNS disorders [15–18]. Hence, using TAT-MeCP2 fusion constructs to replenish intracellular MeCP2 levels may be a promising avenue in treating RTT.

In order to devise such treatment, multiple steps should be undertaken. The appropriate protein constructs have to be successfully recombinantly expressed and purified. The study of stability and aggregation propensity is an important interim step to ensure the proteins’ suitability for downstream experiments. Subsequent tests in cell culture are needed to ensure that the TAT-MeCP2 constructs can penetrate the membrane and the protein can be localized to the appropriate parts of the cell. Then, functional in vitro assays and test injections in control and RTT mice can be performed, TAT-MeCP2 fusion protein penetration into CNS tissues through the BBB demonstrated, with symptoms as well as lifespan monitored and the optimal therapeutic dosage of MeCP2 determined.

This work outlines the expression, purification and characterization of TAT-MeCP2, its eGFP fusion variant (TAT-MeCP2-eGFP), a truncated TAT-MeCP2-eGFP possessing largely just the MBD and NID domains (TAT-minMeCP2-eGFP) as well as their TAT-lacking controls (Fig. 1a). In addition, we present the TAT-mediated uptake of TAT-MeCP2-eGFP and TAT-minMeCP2-eGFP into MeCP2-deficient human dermal fibroblasts and murine NIH3T3 cells via western blots and live cell imaging respectively. Our results provide insight into the initial biophysical and structural properties of these constructs as well as the ability of TAT-MeCP2-eGFP and TAT-minMeCP2-eGFP as compared to their TAT-lacking eGFP control to enter human and mouse fibroblast cells and localize to their various components. Results presented here would serve as an important starting point for further endeavors in the development of protein replacement therapy for RTT.

## Materials and Methods

### Expression of Non-Isotopically Labelled MECP2 Constructs

DNA fragments encoding MeCP2, TAT-MeCP2, MeCP2-eGFP, TAT-MeCP2-eGFP, minMeCP2-eGFP, TAT-minMeCP2-eGFP, TAT-eGFP and eGFP were each cloned into the plasmid pET-28a. Sequences that encode a histidine and strep affinity tags flank the protein-coding sequences on the 5’ and 3’ ends, respectively (Fig. 1a). All constructs were expressed using the same procedure. The plasmid pET-28a containing one of the aforementioned constructs was transformed into the BL21 (DE3) *Escherichia coli* (*E. coli*) expression strain by mixing 1 μl of the former with 50 μl of the latter, electroporating at 2.5 kV, 25 µF, 200 Ohm, addition of 950 μl of Expression Recovery Medium (Lucigen, F98405-1), a one hour incubation at 37 °C shaking at 300 RPM, with subsequent plating of 5 μl of the transformation mixture on LB agar plates which contain 50 μg/ml kanamycin (LB Kan plates). The next day, a single colony was picked from the transformation plate and used to inoculate a 10 ml LB starting culture containing 50 μg/ml kanamycin (LB Kan). This culture was incubated overnight at 37 °C, shaking at 250 RPM. The following day, the overnight culture was used to inoculate a larger, 250 ml LB Kan culture which was incubated at 37 °C, shaking at 250 RPM for five hours. After storage for 30 min at 4 °C, the contents of this culture were used to inoculate eight 270 ml LB Kan cultures with 2.5 g/L meat extract (LB Kan rich), by adding 30 ml to each flask. Protein expression was induced with 1 mM Isopropyl β-d-1-thiogalactopyranoside (IPTG). The cultures were incubated at 30 °C, shaking at 250 RPM for 20 h. The following day, the cells were harvested by centrifugation at 4200 ×* g* at 4 °C for 30 min in a Sigma swinging bucket centrifuge, with subsequent removal of the supernatant. The bacterial cell pellets were stored at −80 °C.

### *Expression of *^*15*^*N-Labelled TAT-MeCP2 and *^*15*^*N-Labelled MeCP2*

The transformation procedure used for ^15^N TAT-MeCP2 and ^15^N MeCP2 was the same as for their unlabelled counterparts (see above). The next day, two clearly distinguished colonies were inoculated into two starting cultures consisting of 50 ml of LB Kan medium. These mixtures were incubated at 37 °C shaking at 250 RPM overnight. The next morning, the cells were pelleted by centrifugation at 4200 ×* g* for 20 min at 4 °C in a Sigma swinging bucket centrifuge. The supernatants were decanted and each pellet was re-suspended in 10 ml of ^15^N minimal growth medium (35 mM Na_2_HPO_4_, 22 mM KH_2_PO_4_, 8.5 mM NaCl, 9 mM ^15^NH_4_Cl, 0.17 mM Ethylenediaminetetraacetic acid (EDTA), 3 μM FeCl_3_ × 6H_2_O, 6 μM ZnCl_2_, 0.76 μM CuCl_2_ × 2H_2_O, 0.60 μM CoCl_2_ × 6H_2_O, 1.62 μM H_3_BO_3_, 0.068 μM MnCl_2_ × 6H_2_O, 0.4% (w/v) ^12^C glucose, 1 mM MgSO_4_, 0.3 mM CaCl_2_, 1 μg/ml biotin, 1 μg/ml thiamine, 50 μg/ml kanamycin). The resulting solutions were in turn used to inoculate two 240 ml ^15^N minimal growth medium cultures grown to an OD_600_ value of 0.8. After half hour storage at 4 °C, protein expression was induced by addition of 1 mM IPTG. The cultures were incubated at 30 °C, shaking at 250 RPM for 20 h. The following day, the cells were harvested by centrifugation at 4200 ×* g* at 4 °C for 30 min in a Sigma swinging bucket centrifuge, with subsequent removal of the supernatant. The cell pellets were stored at −80 °C.

### Purification of MeCP2 and eGFP Constructs

The same purification procedure was employed for all MeCP2 constructs. Multiple bacterial cell pellets were re-suspended in 50 ml of lysis buffer (100 mM Tris–HCl, 500 mM NaCl, 250 mM Urea, 1 mM EDTA, 0.1% (v/v) Triton X-100, 0.1% (v/v)Tween 20, 8% (v/v) Glycerol, pH = 8.0). Additional components which were added to this buffer to their final concentrations of 5 mM β-mercaptoethanol (BME), 50 μg/ml lysozyme, 500 U benzonase, 100 μg/ml phenylmethylsulfonyl fluoride (PMSF) and 500 μl of protease inhibitor cocktail. The re-suspended mixture was incubated on ice for 15 min. The cells were then disrupted by sonication using six 45-s cycles with two-minute pauses. The lysed cells were then centrifuged at 4 °C at 15,000 RPM for 35 min in a Sorval fixed rotor centrifuge to pellet cell debris. The resulting supernatant which contains the cell lysate was filtered through a 0.45 μm filter.

The recombinant proteins were captured from the cell lysate using Strep-Tag affinity chromatography. First, the Strep-Tactin XT column (IBA, 2-5014-001) was equilibrated using 20 ml of the lysis buffer containing 5 mM BME. The cell lysate was then loaded on the column, with the flowthrough collected. The column-bound protein was then washed with 50 ml of high-salt buffer (lysis buffer with a NaCl concentration of 2 M, also containing 5 mM BME), to remove non-specifically bound nucleic acids. Finally, the protein was eluted with 30 ml of elution buffer (lysis buffer with 50 mM D-Biotin, also containing 5 mM BME). The protein was then buffer exchanged into DPBS, 300 mM NaCl, 1 mM Dithiothreitol (DTT), 10% (v/v)_Glycerol, 5% Isopropanol, 0.02% NaN_3_, pH = 7.2 (gel filtration column buffer) using a PD-10 buffer exchange column, and concentrated to a final volume of 2 ml using a 10,000 Molecular Weight Cutoff (MWCO) concentrator, in a Sigma swinging bucket centrifuge at 4 °C. Before proceeding to the next step, the sample was centrifuged in an Eppendorf centrifuge at 10,000 ×* g* at 4 °C, for three minutes to remove any residual precipitated protein.

MeCP2 constructs were then further purified using gel filtration chromatography (GFC). For this purpose, the HiLoad 16/60 S-200 column (Cytiva, 28989335) connected to an AKTA fast performance liquid chromatography (FPLC_ was employed. The protein was loaded on this column which was pre-equilibrated with 120 ml of the gel filtration column buffer. Elution occurred between 57 and 60 ml. A second buffer-exchange into the MeCP2 storage buffer consisting of DPBS, 200 mM NaCl, 10% (v/v) Glycerol, pH = 7.2 was then performed. All constructs were then concentrated to a final volume of 1 ml using a 10,000 MWCO concentrator, in a Sigma swinging bucket centrifuge at 4 °C. To the samples to be used for dynamic light scattering (DLS) and nuclear magnetic resonance (NMR) spectroscopy, 3-[(3-Cholamidopropyl)dimethylammonio]-1-propanesulfonate (CHAPS) was added to a final (w/v) concentration of 0.05%. These samples were flash-frozen and stored at −80 °C.

In addition, removal of lipopolysaccharides (LPS) for TAT-MeCP2-eGFP and TAT-minMeCP2-eGFP samples which were used for western blots and live-cell imaging was carried out. Triton X-114 was added to a final (v/v) concentration of 2.5%. The sample was vortexed and incubated on ice for 30 min and then at 37 °C for 10 min. Centrifugation at 16,000 ×* g* at room temperature for 15 min was the next step, which resulted in the formation of two layers: An aqueous layer containing the protein, and a bottom detergent layer with the separated LPS. The aqueous layer was carefully transferred to another 1.5 ml Eppendorf tube and the extraction was repeated twice to maximize LPS removal. Finally, the aqueous layer was transferred to a Pierce Detergent Removal column (Thermo Scientific, 87779) to eliminate any leftover Triton X-114 and eluted by centrifugation in a Sigma swinging bucket centrifuge at 1,000 ×* g* for 2 min at 20 °C, followed by the addition of CHAPS to a final (w/v) concentration of 0.05%. The samples were flash-frozen and stored at −80 °C. All samples were analyzed on a 12% Sodium Dodecyl Sulphate Polyacrylamide Gel electrophoresis (SDS-PAGE). TAT-MeCP2 was tested for stability by 72-h incubation at 37 °C with subsequent SDS-PAGE analysis. Band intensity was quantified using the Image Lab software.

TAT-eGFP and eGFP were purified in a similar fashion to MeCP2, with two notable differences. The Strep-column bound protein was washed with 50 ml lysis buffer (which contained 500 mM, rather than 2 M NaCl). Following Strep-column chromatography, the samples was buffer-exchanged into DPBS, 10% (v/v) Glycerol, pH = 7.2. No second buffer-exchange step was performed—after elution from the gel filtration column; the proteins were simply concentrated to a final volume of 1 ml using a 10,000 MWCO concentrator, aliquoted and flash-frozen and stored at −80 °C. No LPS extraction was performed on these constructs.

### DLS Experiments

All experiments were performed on the Wyatt Dynapro II DLS Plate Reader (Wyatt) at the Vienna Biocenter Core Facilities. A DLS buffer screen was first performed on TAT-MeCP2-eGFP to determine the optimal buffer conditions for long-term storage. The experiment was then repeated at these optimal conditions on the other MeCP2 constructs. The sample was filtered using a 0.1 µm filter and centrifuged at 10,000 ×* g* for 4 min with the supernatant transferred to another 1.5 ml Eppendorf tube. In a 96 well plate, the protein was mixed with each buffer from the list (Supplementary Table 2) for a final concentration of 0.5 mg/ml. All buffers were DPBS-based at pH = 7.2. The additives tested are NaCl and/or KCl at 200 mM or 400 mM, 1 mM DTT (1 mM) and 0.05% (v/v) Polyethylene Glycol (PEG) 400. The detergents used in the screen are: CHAPS (0.05%), 5-Cyclohexyl-1-Pentyl-β-D-Maltoside (CYMAL®-5, 0.01%), n-Nonyl-β-D-Thiomaltopyranoside (NTM, 0.01%) and *n*-Dodecyl β-D-maltoside (DDM, 0.001%). All detergent percentages are w/v. Silicone oil was put on top of each sample to prevent evaporation and the plate was spun down at 500 ×* g* for 1 min to remove any air bubbles. Measurements were carried out at 25 °C over one week.

For measurements in the optimal buffer, samples were diluted to a final concentration of 0.5 mg/ml with DPBS, 200 mM NaCl, 10% (v/v) Glycerol, 0.05% (w/v) CHAPS, pH = 7.2. The sample was filtered using a 0.1 µm filter and centrifuged at 10,000 ×* g* for 4 min with the supernatant transferred to another 1.5 ml Eppendorf tube (Merck Millipore, Ultrafree-MC-VV Durapore PVDF). The samples were transferred to a 384 well DLS plate. Silicon oil was put on top of each sample to prevent evaporation and the plate was spun down at 500 ×* g* for 1 min to remove any air bubbles. Samples were measured at 37 or 25 °C for 72 h.

### *NMR Spectroscopy of *^*15*^*N TAT-MeCP2 and *^*15*^*N MeCP2*

Fifty microlitres of ^2^H_2_O were added to 500 μl of ^15^N labelled TAT-MeCP2 or ^15^N labelled MeCP2, with the mixture placed into a NMR tube. All experiments were acquired on a Bruker Avance 3 HD + 800 MHz spectrometer at the NMR Facility at the Vienna Biocenter. The acquisition temperature was 25 °C. The 2D ^1^H ^15^N, Transverse relaxation optimized spectroscopy—heteronuclear single quantum correlation (TROSY-HSQC) was performed with the acquisition parameters outlined in Supplementary Table 1. Following the initial experiments, the proteins were stored at 37 °C for 72 h, with the TROSY-HSQC subsequently repeated. NMR data was processed and analyzed using the Topspin 3.5 software.

### Cellular Uptake of Constructs in MeCP2 Deficient Fibroblast Cell Line

A human dermal fibroblast (HDF) cell line from a male RTT patient with neonatal encephalopathy carrying a mutation (c.806delG) in the *MECP2* gene were established as described elsewhere [19]. A single nucleotide deletion in these cells causes a premature stop codon in the *MECP2* reading frame which in turn leads to the expression of an unstable MeCP2 protein, increase in H4K16 acetylation and a RTT-like phenotype [20, 21]. Approval for skin biopsy procurement for research purposes was obtained from the Human Research Ethics Committee of the Children’s Hospital at Westmead, Australia.

Human dermal fibroblasts were cultured in DMEM (Gibco, 41,966) supplemented with 20% FBS (Fetal bovine serum, Sigma, F9665) and 1% Penicillin–Streptomycin (Gibco, 15140122) at 37 °C, under a humidified atmosphere of 5% CO_2_. For uptake studies with TAT-MeCP2-eGFP, TAT-minMeCP2-eGFP and eGFP, 1.5 × 10^6^ of the aforementioned MeCP2-deficient cells were plated on 100 mm dishes (664,160, Greiner) and left to attach overnight before adding 500 nM of each protein sample and incubating for one hour. The cells were then washed three times with 0.5 mg/ml heparin in DPBS to remove external membrane-bound protein. For cytosolic and nuclear protein extraction, cells were harvested and washed twice with ice-cold DPBS, centrifuged at 500 ×* g* for 5 min and the protein was extracted from adherent cells as described [22] with slight modifications. For western blotting, 10 μg of each protein were run on 12% acrylamide gels, followed by transfer to a nitrocellulose membrane using the iBlot® Dry Blotting system (Invitrogen, IB1001). Blocking was performed with 5% milk powder in 0.05% Tween-20 in PBS (PBS-T) for 1 h at room temperature. The primary antibodies used were GFP (abcam, ab290), H3 (Milipore, 06–755) and β-tubulin (Sigma, T4026), all in 1:1,000 dilution. Incubation with primary antibodies was carried out in 5% milk powder in PBS-T, overnight rotating at 4 °C. The next day, incubation with mouse (Life Technologies, 7076S) and rabbit (Life Technologies, 7074S) secondary antibodies was performed at 1:10,000 dilution as appropriate, also in 5% milk powder in PBS-T, for 1 h at room temperature. The blot was imaged using Clarity Western ECL Substrate (Bio-Rad, 1,705,061) according to the manufacturer’s instructions and the ChemiDoc Touch Imaging System.

### Live Cell Imaging

A total of 20,000 murine NIH3T3 cells per well, were seeded a day in advance on 8-well collagen IV treated µ-slides, (ibidi, 80822). NIH3T3 cells were cultured in DMEM (Gibco, 41966) supplemented with 10% FBS (Fetal bovine serum, Sigma, F9665) and 1% Penicillin–Streptomycin (Gibco, 15140122) at 37 °C, under a humidified atmosphere of 5% CO_2_. On the day of the experiment, the medium was removed and 5 µM of each, of TAT-MeCP2-eGFP, TAT-minMeCP2-eGFP, TAT-eGFP and eGFP had been diluted in culture medium and applied directly to the cells for one hour. Following incubation, 1.5 μM Hoechst 33342 (Thermo Scientific) was added for 5 min to counterstain the nucleus. The cells were then washed with DPBS, then with 0.5 mg/ml heparin in DPBS to remove external membrane-bound protein followed by two final washing steps with DPBS. Subsequently, the cells were incubated with SCM143-1 Bright Cell MEMO® Photostable media (Millipore) and immediately used for live cell imaging on a confocal microscope (Leica TCS SP8) equipped with temperature and gas control (37 °C, 5% CO_2_).

### Cell Viability Assessment Assay

A 3-(4,5-dimethylthiazol-2-yl)-2,5-diphenyltetrazolium Bromide (MTT) assay was performed as previously described [23] with slight modifications. On the day before treatment, NIH3T3 cells were seeded in a 96-well-plate (25,000 cells per well, Greiner, 655,180). After treatment with the various protein constructs for one hour, the medium was removed and the cells incubated with 110 µL medium containing 455 µg/ml MTT for 2 h. Then, 110 µl extraction buffer (20% sodium dodecyl sulfate and 50% N,N-dimethylformamide, pH = 4.7) was added to each well following a final incubation overnight at 37 °C. Absorbance was measured at 570 nm using Biotek plate reader. Absorbance values were normalized to untreated cells.

## Results

### MeCP2 Expression, Purification and Initial Characterization

All MeCP2 constructs were purified using Strep-Tactin affinity chromatography from the lysed *E. coli* media and GFC as a second, polishing, purification step. GFC omission significantly compromises downstream TAT-MeCP2 stability, with less than 40% of intact protein remaining following an incubation period of 72 h at 37 °C (Supplementary Fig. 1). During GFC, the full length constructs travelled through the column significantly faster compared to what was to be expected for proteins of their size, as their elution volume was between 56.5 and 59.0 ml (Supplementary Fig. 2). This would correspond to a protein with a molecular weight between 300 and 400 kDa, while the molecular weights of the (TAT-)MeCP2 constructs is 58.6 kDa and 83.3 kDa for (TAT-)MeCP2-eGFP variants. Similar behavior was observed on SDS-PAGE: (TAT-)MeCP2 constructs were shown to migrate between 72 and 95 kDa, while (TAT-)MeCP2-eGFP appear between 95 and 130 kDa (Fig. 1c). The likely cause for such behavior is the high abundance of proline residues in the C-terminal portion of the MeCP2 sequence (Fig. 1b, marked in bold) which impart behavior consistent with that of an elongated molecule, a phenomenon that has been previously observed [24, 25]. (TAT-)minMeCP2-eGFP constructs, on the other hand, which do not contain a region of the protein which is rich in proline residues migrated in line with their molecular weight, both during GFC and on the SDS-PAGE (Fig. 1c, TAT-minMeCP2-eGFP, Supplementary Fig. 2).

### Stability and Aggregation Propensity Analysis of MeCP2 Constructs

To identify optimal buffer conditions for the MeCP2 constructs, a DLS buffer screen was performed. The protein was incubated in a 96 well-plate with each well filled with 36 different buffer compositions. Initial intensity and radius measurements were recorded at the start of the experiment with final respective measurements obtained after 3-day incubation at 25 °C. Throughout the experiments, two parameters were monitored: Normalized intensity and radius. Both parameters (especially the radius) serve as readout for protein stability and aggregation propensity. Their initial values are indicative of the size of the protein at the beginning of the assay. Abnormally high radius values indicate the presence of high molecular weight aggregates. Correspondingly, final intensity values provide information regarding protein stability and the end of the experiment, where significant intensity decreases, indicate protein degradation over time. Final radius indicates the size of the protein at the end of the experiment. Increases in radius suggest formation of high molecular weight aggregates during the incubation period, decreases in radius occur due to a reduction in soluble protein levels either due to degradation or insoluble aggregation over time. An ideal buffer should be one in which the protein is both stable and not aggregation-prone. In such a buffer, therefore, the initial radius would be close to the predicted radius of MeCP2, as well as final intensity and radius values that are as close as possible to their initial counterparts (i.e., the final/initial intensity and radius ratios should be close to 1).

TAT-MeCP2-eGFP was found to exhibit a high degree of stability and little aggregation propensity over one week at 25 °C in the presence of the zwitterionic detergent CHAPS or other mild maltoside detergents such as CYMAL-5, NTM or DDM in the DPBS-based buffer. This is evidenced by consistent intensity and hydrodynamic radius values over time, the ratio of which was close to 1 (Supplementary Table 2). The initial hydrodynamic radius of TAT-MeCP2-eGFP was found to be significantly higher than what is expected for a globular protein of the same size. However, this value was found to be in line with what previous studies, consistent with MeCP2’s IDP properties [26]. Out of all the buffer conditions, DPBS, 200 mM NaCl, 10% (v/v) Glycerol, 0.05% (w/v) CHAPS, pH = 7.2 (Supplementary Table 2, in bold) was picked for further structural analysis as well as for western blotting and live-cell imaging, due to its best suitability for the cellular incubations which precede the latter two experiments.

To ascertain that the other MeCP2 constructs would behave in a similar manner in this buffer system, MeCP2, MeCP2-eGFP, minMeCP2-eGFP TAT-MeCP2, TAT-MeCP2-eGFP and TAT-minMeCP2-eGFP were subjected to a DLS stability test at both 37 °C and 25 °C for 72 h in DPBS, 200 mM NaCl, 10% (v/v) Glycerol, 0.05% (w/v) CHAPS, pH = 7.2. All constructs remained largely stable and did not exhibit any significant signs of aggregation over the aforementioned time period at 37 °C (Table 1) as well as at 25 °C (Table 2) as indicated by the ratio of their initial and final intensity and radius measurements being close to 1. The stability of MeCP2-eGFP at 25 °C decreased somewhat more significantly compared to other constructs. Similar to TAT-MeCP2-eGFP, the radii of all full-length MeCP2 constructs were abnormally large for a globular protein of the corresponding size. The minMeCP2-eGFP and TAT-minMeCP2-eGFP were also found to be stable and aggregation-free in the same buffer at both 37 °C (Table 1, in bold) and 25 °C (Table 2, in bold) for 72 h. Their radii, however, were found to be smaller, due to the absence of some of MeCP2’s disordered portions. There was no significant difference in the radii of the MeCP2 constructs and their GFP fusion derivatives. The TAT-containing full-length proteins were found to possess a noticeably larger radius compared to their TAT-lacking counterparts, indicating that the presence of this 11-amino acid stretch imparts an even further extended conformation. A similar TAT-dependent increase in size was not found, however, in the minimal constructs. The radii of full-length TAT-MeCP2-eGFP, and minimal (TAT-)MeCP2-eGFP were higher at 37 °C than 25 °C. On the other hand, however, the radii of other constructs experience a decrease with increasing temperature. Hence, no clear effect of temperature on construct size has been established.

**Table 1:** Protein intensity and radius values before and after a 72 h incubation at 37 °C, DPBS, 200 mM NaCl, 10% (v/v) Glycerol, 0.05% (w/v) CHAPS, pH = 7.2. The TAT-minMeCP2-eGFP and minMeCP2-eGFP constructs are marked in bold

Construct, 37 °C	Parameter	Initial value	Final value	Ratio
MeCP2	Intensity	4.1 * 10^6^ Cnt/s	4.3 * 10^6^ Cnt/s	1.05
	Radius	6.3 nm	6.1 nm	0.97
TAT-MeCP2	Intensity	5.3 * 10^6^ Cnt/s	6.7 * 10^6^ Cnt/s	1.26
	Radius	7.7 nm	7.5 nm	0.97
MeCP2-eGFP	Intensity	4.4 * 10^6^ Cnt/s	4.6 * 10^6^ Cnt/s	1.05
	Radius	6.7 nm	6.4 nm	0.96
TAT-MeCP2-eGFP	Intensity	6.5 * 10^6^ Cnt/s	6.4 * 10^6^ Cnt/s	1.00
	Radius	9.1 nm	8.0 nm	0.89
**TAT-minMECP2-eGFP**	**Intensity**	4.2*10^**6**^** Cnt/s**	4.7*10^**6**^** Cnt/s**	**1.12**
	**Radius**	**5.2 nm**	**5.2 nm**	**1.05**
**minMECP2-eGFP**	**Intensity**	4.2*10^**6**^** Cnt/s**	3.7*10^**6**^** Cnt/s**	**0.95**
	**Radius**	**5.5 nm**	**4.9 nm**	**0.90**

**Table 2:** Protein intensity radius values before and after a 72 h incubation at 25 °C, DPBS, 200 mM NaCl, 10% (v/v) Glycerol, 0.05% (w/v) CHAPS, pH = 7.2. The TAT-minMeCP2-eGFP and minMeCP2-eGFP constructs are marked in bold

Construct, 25 °C	Parameter	Initial value	Final value	Ratio
MeCP2	Intensity	2.5 * 10^6^ Cnt/s	2.1 * 10^6^ Cnt/s	0.85
	Radius	7.2 nm	7.0 nm	0.97
TAT-MeCP2	Intensity	2.8 * 10^6^ Cnt/s	1.8 * 10^6^ Cnt/s	0.64
	Radius	8.2 nm	8.3 nm	1.01
MeCP2-eGFP	Intensity	3.0 * 10^6^ Cnt/s	2.0 * 10^6^ Cnt/s	0.67
	Radius	6.9 nm	6.3 nm	0.91
TAT-MECP2-eGFP	Intensity	3.3 * 10^6^ Cnt/s	2.81 * 10^6^ Cnt/s	0.85
	Radius	8.1 nm	7.8 nm	0.96
**TAT-minMECP2-eGFP**	**Intensity**	2.8*10^**6**^** Cnt/s**	2.6*10^**6**^** Cnt/s**	**0.94**
	**Radius**	**4.2 nm**	**4.2 nm**	**1.00**
**minMECP2-eGFP**	**Intensity**	4.1*10^**6**^** Cnt/s**	4.8*10^**6**^** Cnt/s**	**1.17**
	**Radius**	**4.4 nm**	**4.1 nm**	**0.93**

### NMR Spectroscopy of TAT-MeCP2 and MeCP2

**Fig. 2:**
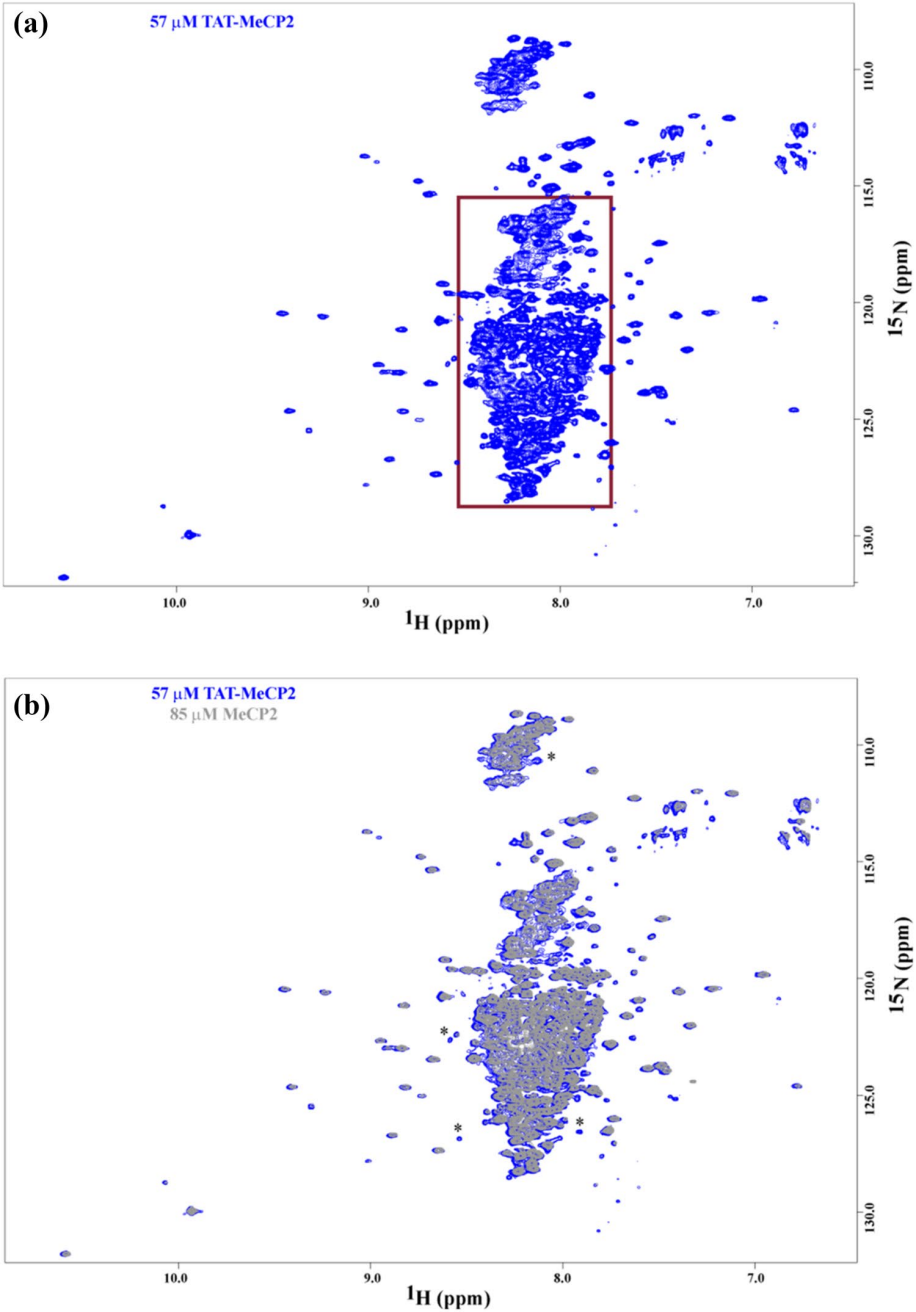
NMR Spectroscopy of TAT-MeCP2 and TAT-MeCP2 **a** A 2D ^1^H, ^15^N TROSY- HSQC of ^15^N labelled TAT-MeCP2. Signals originating from disordered portions of the protein are largely localized to the boxed area of the spectrum. These are flanked by cross-peaks purported to emanate from the structured parts of the protein. **b** An overlay of 2D ^1^H, ^15^N HSQC spectra ^15^N labelled TAT-MeCP2 (marked in blue) and ^15^N labelled MeCP2 (marked in grey). Representative non-overlapping cross-peaks are marked with an asterisk. All spectra were acquired on a Bruker Avance 3 HD + 800 MHz spectrometer at 25 °C

To gain additional insight into the structural and conformational properties of MeCP2, NMR spectroscopy was employed. In the 2D ^1^H, ^15^N TROSY-HSQC of isotopically labelled ^15^N TAT-MeCP2 (Fig. 2a) and ^15^N MeCP2, a large cluster of cross-peaks between 7.8 and 8.8 ppm (boxed in red) is observed. These signals largely correspond to the protein’s disordered components, which should comprise the largest portion of the MeCP2 sequence. In addition to this cluster, well-dispersed individual cross-peaks are observed in regions flanking it. These peaks most likely correspond to the structured portions of MeCP2, that is, the MBD and the NID. These findings indicate that both unstructured as well as structured components are present in both MeCP2 constructs.

The TROSY-HSQC spectra of ^15^N TAT-MeCP2 and ^15^N MeCP2 were superpositioned (Fig. 2b) for comparison. While some signals present in the TAT-MeCP2 spectrum are missing in its MeCP2 counterpart (possibly those which account for the TAT residues in the TAT-MeCP2 construct), a high degree of overlay in the well-resolved signals which likely correspond to the structured parts of MeCP2 is apparent. This serves as indication that the presence of TAT does not impart a significant conformational change on the structured MeCP2 components in TAT-MeCP2. A very high degree of spectral overlap has precluded a more rigorous NMR analysis of either one of those constructs.

To investigate TAT-MeCP2 and MeCP2 stability over time, both samples were incubated at 37 °C for 72 h, with the NMR experiments repeated. While some degradation indeed occurs, as evidenced by the appearance of signals boxed (Supplementary Fig. 3a and b), the predominant absence of chemical shift changes, including in the well resolved, structured signals after the incubation, indicates a significant degree of stability for both TAT-MeCP2 and MeCP2 over time. Markedly less degradation has been observed in MeCP2 compared to its TAT-MeCP2 counterpart.

### Transduction of TAT-MeCP2-eGFP and TAT-minMeCP2-eGFP into MeCP2-Defficient Cells

To study TAT-mediated transduction of MeCP2 fusion variants, two of these constructs, TAT-MeCP2-eGFP and Tat-minMeCP2-eGFP were each incubated with MeCP2-deficient cells with uptake levels subsequently analyzed by western blotting. For this purpose, the HDF cell line lacking MeCP2 was employed.

**Fig. 3:**
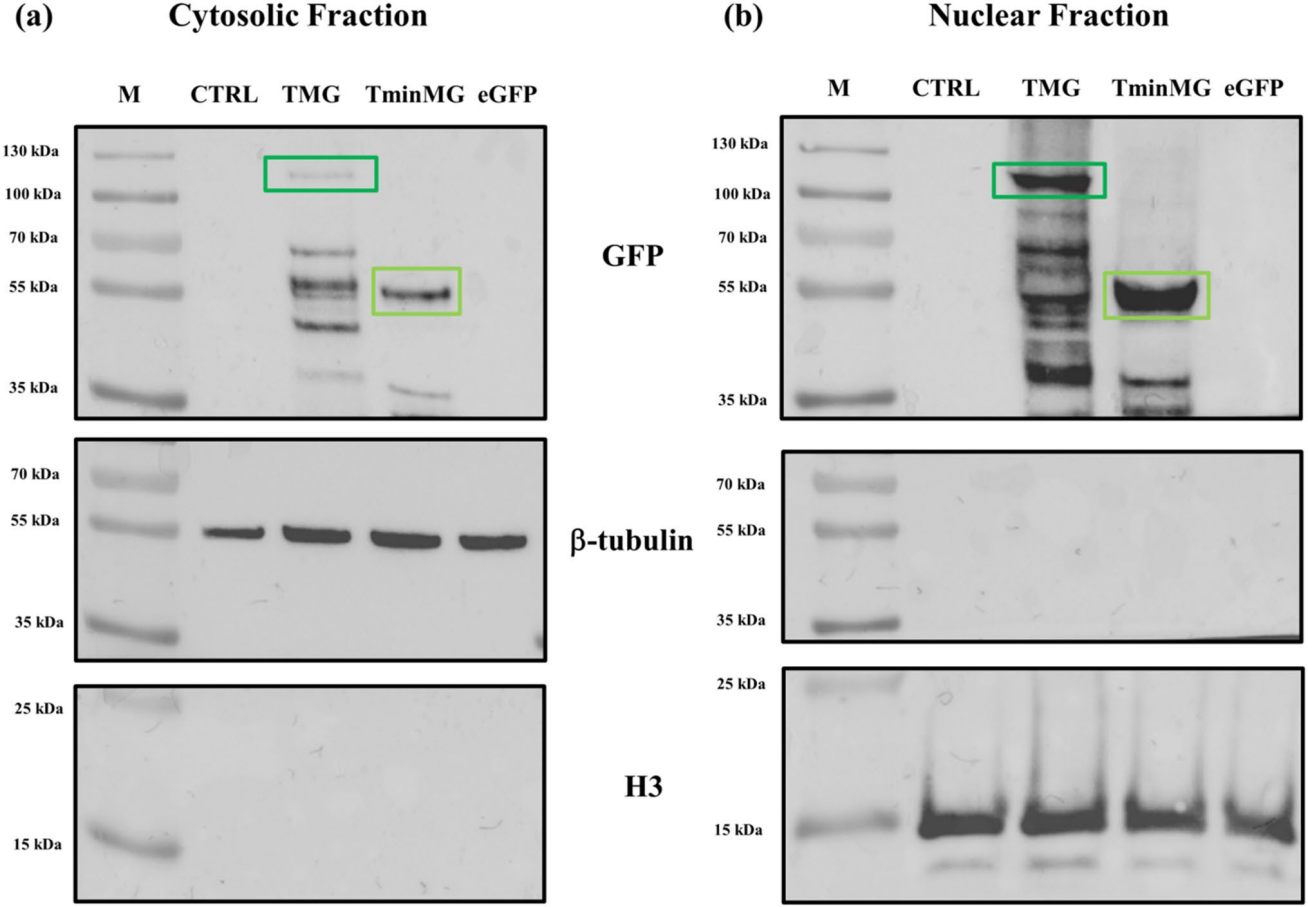
Uptake of TAT-MeCP2-eGFP and TAT-minMeCP2-eGFP into **a** Cytosolic fraction; **b** Nuclear fraction of c.806delG cells. M–Marker, CTRL–Cells without protein, TMG–Cells incubated with TAT-MeCP2-eGFP (boxed in dark green), TminMG–Cells incubated with TAT-minMeCP2-eGFP (boxed in light green), eGFP–Cells incubated with eGFP, without TAT. ß-tubulin and H3 were used as the cytosolic and nuclear markers respectively

Both TAT-MeCP2-eGFP and TAT-minMeCP2-eGFP have shown to successfully transduce into male hemizygous c.806ldelG fibroblasts and predominantly localize to the nucleus (Fig. 3b), compared to the cytoplasm (Fig. 3a), as both of these constructs possess nuclear localization signals (Fig. 1b). Markedly higher uptake levels of TAT-minMeCP2-eGFP compared to its full-length counterpart were observed. This construct has also demonstrated a higher degree of stability compared to TAT-MeCP2-eGFP. In contrast, no uptake was observed in the case of the eGFP. These findings demonstrate the utility of the TAT sequence in successfully transducing TAT-MeCP2-eGFP variants into RTT-patient cells.

### Live-Cell Imaging of TAT-MeCP2-eGFP and TAT-minMeCP2-eGFP

**Fig. 4:**
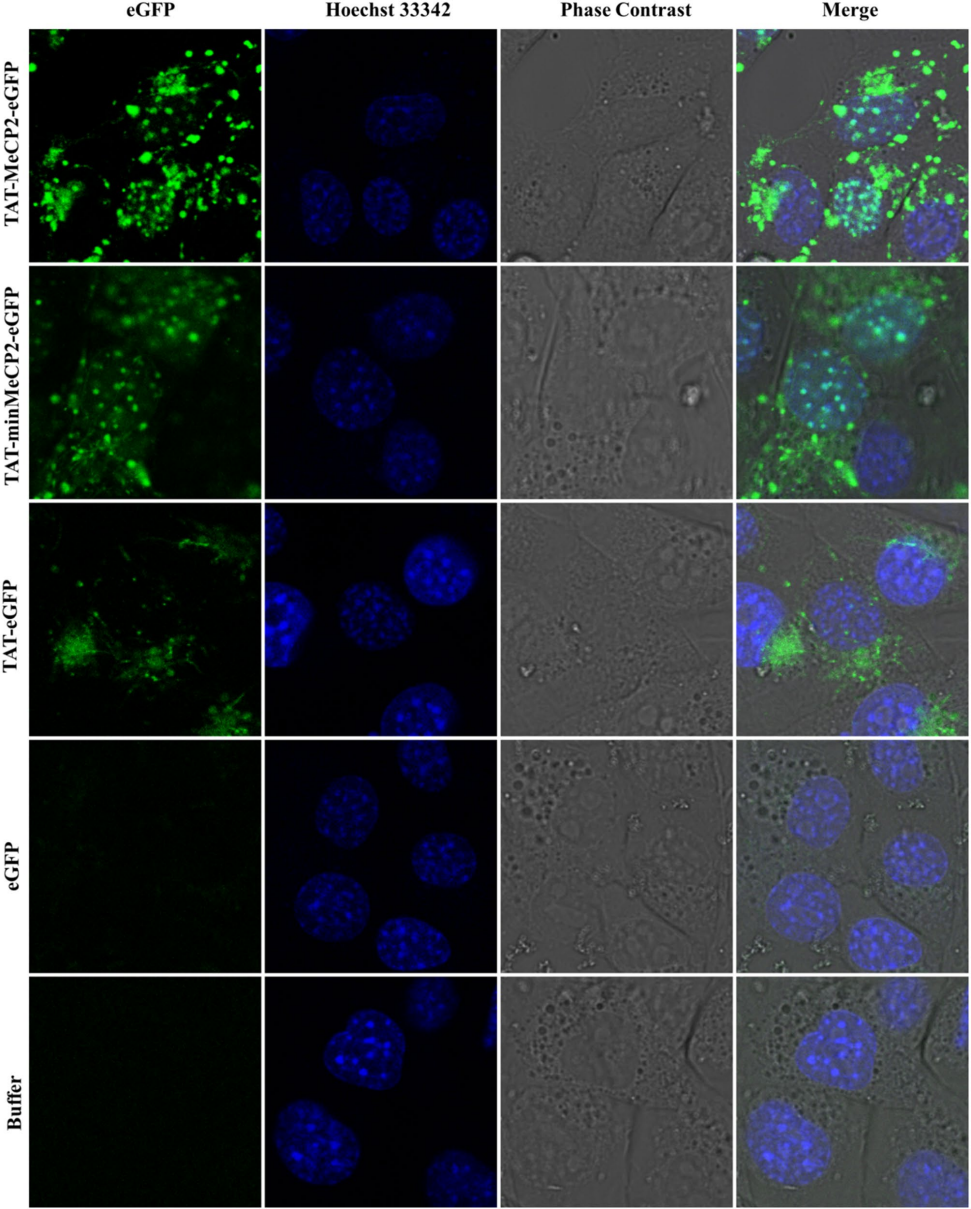
Uptake of TAT-MeCP2-eGFP, TAT-minMeCP2-eGFP, and TAT-eGFP into living cells. Murine embryonic fibroblast NIH3T3 cells were incubated with 5 μM of (top to bottom) TAT-MeCP2-eGFP, TAT-minMeCP2-eGFP, TAT-eGFP and eGFP for one hour. Chromatin distribution was visualized in living cells with Hoechst 33342 (blue). Cells treated with buffer are also shown (bottom panel). Confocal images depict representative snapshots of living cells in the phase contrast image

Uptake efficiency and cellular distribution of full length and minimal TAT-MeCP2-eGFP fusion protein variants into living cells was also investigated by live-cell imaging in murine NIH3T3 cells. Recent reports have shown that MeCP2 is accumulated in heterochromatic foci of these cells [27, 28]; therefore, this experiment serves as a good model to study intra-cellular distribution patterns of the aforementioned constructs. One-hour incubation with 5 μM of each TAT-MeCP2-eGFP and TAT-minMeCP2-eGFP with subsequent heparin treatment and Hoechst 33342 counterstaining, has revealed localization mainly to heterochromatic chromocenters in NIH3T3 nuclei with some additional protein presence in the cytoplasm (Fig. 4, first and second panel, respectively). Clear cytoplasmic localization is observed, as expected, for TAT-eGFP, a protein which does not possess a nuclear localization signal (Fig. 4, third panel). We also used recombinant eGFP protein for a control transduction experiment which showed no uptake after incubation for the same period of time (Fig. 4, fourth panel). No apparent morphological changes have been observed in the protein-treated cells upon sample addition (Fig. 4, fifth panel) and a MTT assay revealed no decrease in cell survival compared to the controls (Supplementary Fig. 4), strongly indicating that cell viability is not compromised by recombinant protein presence. Taken together, these findings demonstrate that the transduction efficiency of TAT-MeCP2-eGFP and TAT-minMeCP2-eGFP proteins is high in living mouse embryonic fibroblasts and provides evidence that this strategy is useful to transport exogenously applied MeCP2 protein variants across cell membranes and into their nuclei.

## Discussion

RTT is a debilitating, neurodevelopmental disorder, largely caused by the loss of proper function of the transcriptional regulator MeCP2 [1]. One possible treatment option is to restore functioning MeCP2 levels in the cell by administering this protein exogenously, while fused to the CPP TAT. To study the potential feasibility of such an approach, we have heterologously expressed, purified, and performed cellular uptake studies on a number of MeCP2 constructs. Since sample stability is of great importance for these experiments, a screen was carried out to search for optimal buffer conditions. As MeCP2 is an IDP, with disordered portions accounting for the majority of its sequence, DLS is a suitable technique that can be used to gain insight into the stability and aggregation propensity of this protein and how they change over time via intensity and radius readouts—a necessary prerequisite for downstream cellular incubation experiments.

TAT-MeCP2-eGFP displayed a high degree of stability and showed no signs of aggregation, both at the start of the incubation and over one week in DPBS, 200 mM NaCl, 10% (v/v) Glycerol, 0.05% (w/v) CHAPS, pH = 7.2. This is indicated by an initial hydrodynamic radius which was in line with previous findings [26], which changed little over the incubation period. Similar behavior is observed with other additives and detergents (Supplementary Table 2); however it was decided to proceed with the aforementioned conditions as those were deemed most favorable for subsequent cellular incubations for western blots and live-cell imaging experiments.

When incubated in DPBS, 200 mM NaCl, 10% (v/v) Glycerol, 0.05% (w/v) CHAPS, pH = 7.2 over 72 h, all other MeCP2 constructs also displayed high degree of stability and showed no signs of aggregation both at 37 °C (Table 1) and 25 °C (Table 2). As mentioned, the full-length MeCP2 as well as their eGFP fusion variants were found to possess an abnormally large hydrodynamic radius for a globular protein of their size, consistent with previous studies [26]. Other work has shown that while the structured MeCP2 domains such as the MBD possess a radius consistent with that of globular protein, a MeCP2 protein construct that harbors this domain along with its disordered flanking regions, has a very significantly enlarged radius of 5.5 nm [29]. This further shows the degree of disorder that the random coil portions of MeCP2 impart on structured domains such as well as on the overall protein conformation.

Another feature exhibited by all full-length MeCP2 constructs is mobility in GFC and on the SDS-PAGE that is not consistent with their molecular weight—migration patterns expected for a protein much larger than their actual size are observed (Fig. 1c, Supplementary Fig. 1, Supplementary Fig. 2). This behavior is not seen in the minimal (TAT-)minMeCP2-eGFP (Fig. 1c, Supplementary Fig. 2), constructs even though their radius is still larger than what it is expected for a globular protein (Tables 1 and 2, marked in bold). There are still disordered portions in these proteins that would account for their markedly enlarged size. But those portions are evidently not responsible for the aberrant GFC and SDS-PAGE migration. The full-length MeCP2 contains a polyproline stretch (Fig. 1b, marked in bold), which is absent in the minimal MeCP2-eGFP constructs. Polyproline stretches, which are known to be present in a number of proteins, possess a number of unique characteristics which include: Structural rigidity, resistance to compaction, which in turn may lead to abnormal migration in SDS-PAGE and GFC [24, 25, 30, 31]. As such, it is likely that MeCP2’s polyproline stretch is the portion of the protein which is responsible for its abnormal SDS-PAGE and GFC mobility.

The polyproline stretch is located in a region of MeCP2 that it thought to be involved in Group 2 WW splicing factor binding, and frameshift mutations (or truncations) there account for about 10% of RTT cases [32]. At the same time, substitution mutations do not lead to severe phenotypes, and neither does a complete deletion of the region as is the case in minMeCP2-eGFP construct [11]. It is plausible that frameshifts as well as partial truncations introduce a high degree of overall instability in MeCP2 which may lead to its partial degradation and a severe misregulation of its function, while complete binding domain deletion only results in a less severe functional misregulation, independent of splicing factor binding. Alternatively, MeCP2 frameshifts may alter protein solubility that may compromise chromatin phase transition which MeCP2 is known to be implicated in [27], which in turn may also lead to RTT pathology. Finally, if the mutated *MECP2* mRNA containing the aforementioned frameshift mutation is degraded prior to being translated, this will lead to a MeCP2-null phenotype and consequentially RTT pathology.

In addition, polyproline motifs are also known to inhibit nascent polypeptide translation due to ribosomal stalling [33]. As such, one can speculate whether the polyproline stretch in MeCP2 is responsible for attenuating its own expression levels. This hypothesis may be supported by the higher overall *E. coli* expression yields of the TAT-minMeCP2-eGFP and minMeCP2-eGFP compared to their full-length counterparts (data not shown). On the other hand, previous work shows that amounts of minMeCP2-eGFP in the appropriate mouse knockout line were lower compared to its wild type form [11] and other factors, such as the presence or absence of various post-translational MeCP2 modifications in eukaryotic cells, may be at play when it comes to protein stability. Hence, no clear conclusion can be drawn on this matter at this time.

The full length TAT-MeCP2-(eGFP) constructs were shown to possess a markedly higher radii compared to their TAT-missing counterparts. Whether this difference is unique to full-length MeCP2 or is a more general property of TAT-fusion proteins, remains unclear. All full-length MeCP2 constructs with the exception of TAT-MeCP2-eGFP have exhibited a decrease in their hydrodynamic radii at 37 °C compared to 25 °C. It has been generally shown that IDPs, somewhat counterintuitively, actually contract with increasing temperature, likely due to the destabilization of extended, locally ordered structural components [34–36]. However a direct relationship of IDP size to temperature has, albeit rarely, also been described [37].

Isotopically ^15^N-labelled MeCP2 and TAT-MeCP2 were also studied by NMR spectroscopy. As NMR of IDPs is complicated by low chemical shift dispersion [38, 39], which results in difficulty in spectral interpretation, our analysis was limited to a 2D ^1^H, ^15^N TROSY-HSQC to determine whether any signals which may correspond to structured MeCP2 portions would be present as well as whether the presence of TAT imparts any significant changes in those signals. Indeed, in addition to the clustered, narrow linewidth cross-peaks between 8.0 and 8.5 ppm, a number of well-resolved signals with wider linewidth upfield and downfield in the ^1^H dimension are observed (Fig. 2a). These signals with a high degree of likelihood correspond to the structured portions of MeCP2, namely the MBD and the TRD. Previous work has also shown that well-dispersed, MBD-corresponding cross-peaks are present in the full-length MeCP2 2D ^1^H, ^15^N TROSY-HSQC spectra [40]. When MeCP2 and TAT-MeCP2 spectra are overlaid, no significant changes in the chemical shifts of those signals are observed, an indication that the TAT-peptide does not exert any visible conformational changes to these regions of MeCP2.

As it was now apparent that the purified constructs are stable and aggregation-free over a period of at least 72 h, we then proceeded to utilize two of these constructs to investigate TAT-mediated transduction of MeCP2. For this purpose, we employed western blotting and live-cell imaging using TAT-MeCP2-eGFP and TAT-minMeCP2-eGFP. These two techniques have been used on numerous occasions to study CPP uptake [41–44]. When studying the cellular transduction of CPPs, a number of considerations are important. First, membrane-bound TAT-fusion protein that has not been transduced has to be removed in both experiments. The glycosaminoglycan heparin, which is known to act as a competitive inhibitor of TAT binding with the cell surface, was previously successfully employed for this purpose [45, 46]. Indeed, when used here, very little to no adhering protein is observed on the cell membrane (Supplementary Fig. 5). In addition, care also has to be taken to avoid methods that result in misleading data. For instance, it has been shown that fixation techniques with subsequent fluorescence microscopy analysis, generate abnormal CPP localization patterns and hence cannot be used [47]. In live-cell imaging, cells are not fixed; hence protein localization can be accurately investigated and assessed, which is why this technique (and not immunofluorescence staining with fixation) was used to visualize intracellular TAT-MeCP2-eGFP and TAT-minMeCP2-eGFP following their uptake.

Western blots of c.806delG cells treated with TAT-MeCP2-eGFP as well as with TAT-minMeCP2-eGFP have revealed uptake of both constructs into the cells, with the largest protein amounts being observed in the nucleus and smaller amounts present in the cytoplasm. Predominantly nuclear compartmentalization occurs due to the presence of nuclear localization signals in both constructs. In contrast, no uptake was observed for eGFP. TAT-minMeCP2-eGFP was found to be uptaken more readily compared to its full-length counterpart. This increased uptake efficiency may be ascribed to higher TAT-minMeCP2-eGFP stability compared to TAT-MeCP2-eGFP during cellular incubation. Alternatively, the IDP portions in the full-length MeCP2 construct may adhere to the cell membrane and in turn inhibit TAT-mediated transduction.

Both the full-length TAT-MeCP2-eGFP as well the TAT-minMeCP2-eGFP constructs were also found to be readily taken up into murine NIH3T3 cells compared to eGFP negative control. As in the case of our western blot findings, both constructs were found to localize to the nucleus, particularly, to heterochromatic condensates. This kind of heterochromatic accumulation is in line with previous findings which link MeCP2 to heterochromatic condensate modulation in mouse embryonic cells which plays a role in neuronal maturation that also carries clinical implications [27, 28].

This study has a number of limitations. First, the functional implications of TAT-MeCP2-eGFP and TAT-minMeCP2-eGFP transduction have not been investigated yet. One such study would seek to determine whether the uptake of the above mentioned constructs could lead to potential reversal of histone hyperacetylation, a hallmark of RTT on the cellular level [48], and whether any differences in such putative reversal exist between the full-length and the minimal constructs. In addition, the exact MeCP2 dosage necessary for a successful rescue has not been determined. When replenishing functional MeCP2, care must be taken not to exceed this amount, as overdose may lead to severe symptoms associated with *MECP2* duplication syndrome [49]. To accurately quantify which MeCP2 levels would correlate with the desired therapeutic effect in mice, methods such as an electrochemiluminescence based assay (ECLIA) could be utilized [50, 51]. Finally, proof-of-principle experiments, which would clearly demonstrate the penetration of TAT-MeCP2 constructs across the BBB into the cells of the CNS and their effect on the symptoms and lifespan in Rett mouse models, would be critical for unambiguously establishing the benefit of protein replacement therapy. Increasing numbers of publications in animal models demonstrate the efficiency of this technology for the potential treatment of other neurological disorders. Such studies have already been recently published for Alzheimer disease [52], purine nucleoside phosphorylase deficiency [53], Friedreich ataxia [44] and very recently cyclin-dependent kinase-like 5 (CDKL5) Deficiency Disorder [18], a disorder that shares similar symptomatic features with RTT.

In conclusion, expression and purification of MeCP2 constructs tethered to the CPP TAT, their TAT-lacking controls as well as their full-length and minimal eGFP variants were successfully performed. All constructs were found to be largely stable and non-aggregating over 72 h and were hence deemed suitable for downstream uptake experiments. Unlike the full-length MeCP2 constructs, minMeCP2-eGFP did not exhibit aberrant GFC and SDS-PAGE migration, likely due to the absence of a polyproline stretch in their sequence. Western blots and live-cell imaging of transduced TAT-MeCP2-eGFP and TAT-minMeCP2-eGFP, clearly demonstrate the utility of this CPP in transducing these MeCP2 fusion proteins which subsequently localize to the nucleus. These findings are an important step in demonstrating the utility of protein replacement therapy in treating RTT and serve as a starting point for ongoing and future work in this field.

## Supplementary Information

Below is the link to the electronic supplementary material.

Supplementary file1 (DOCX 2435 KB)

## Data Availability

Datasets generated during and/or analysed during the current study are not publicly available but can be provided from the corresponding author upon reasonable request.
