# Supplementary material for: Expression, Purification, Characterization and Cellular Uptake of MeCP2 Variants
Source: Protein J. 2022 May 12;41(2):345–59. doi: 10.1007/s10930-022-10054-9 (PMC9122891; doi:10.1007/s10930-022-10054-9)
Supplement: Supplementary file 1 — Supplementary file1 (DOCX 2435 KB) [file 10930_2022_10054_MOESM1_ESM.docx]

**Supplementary Information**


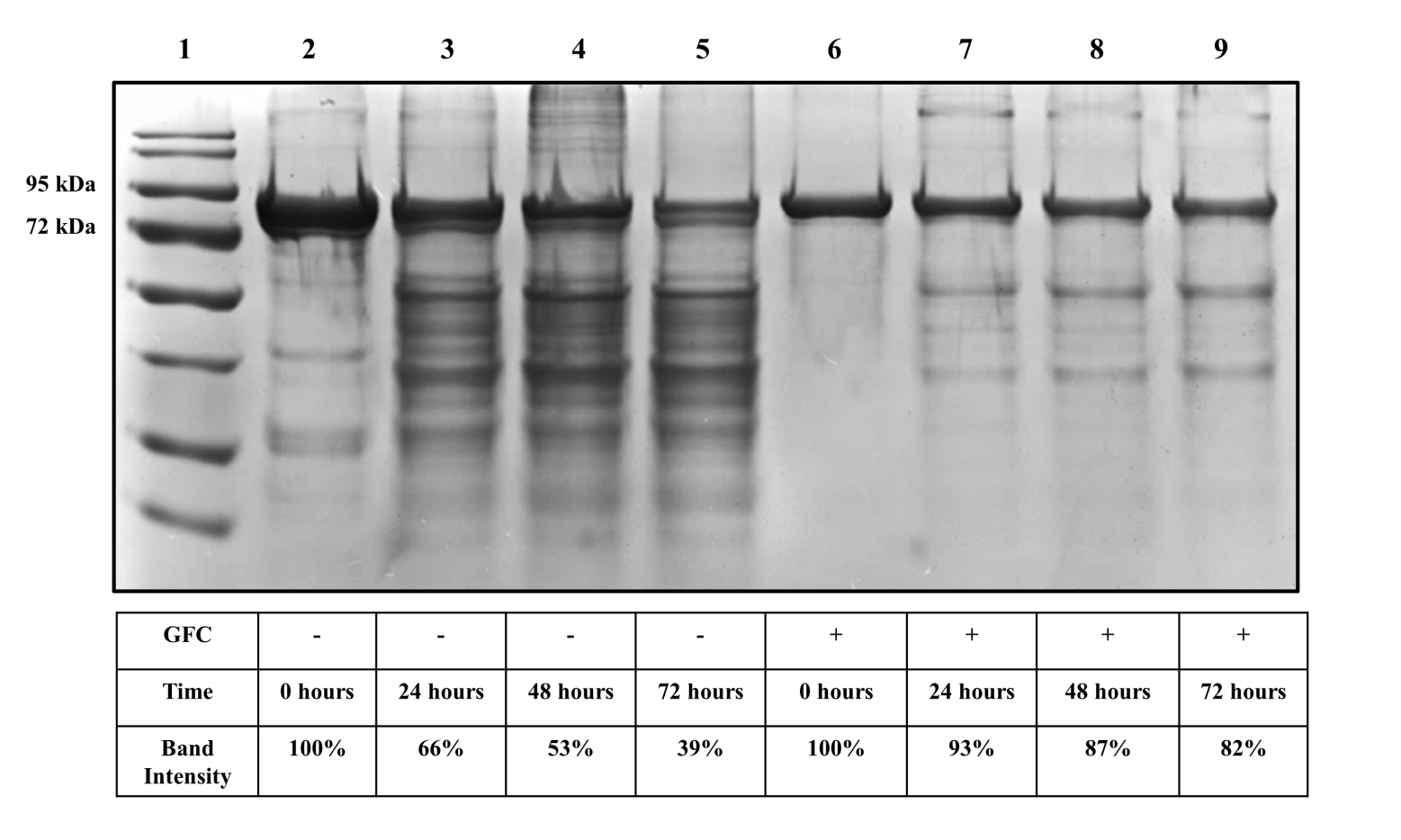


**Supplementary Fig. 1** – TAT-MeCP2 stability test at 37 °C during 24, 48 and 72 hours without (-) and with (+) GFC as a purifying step.


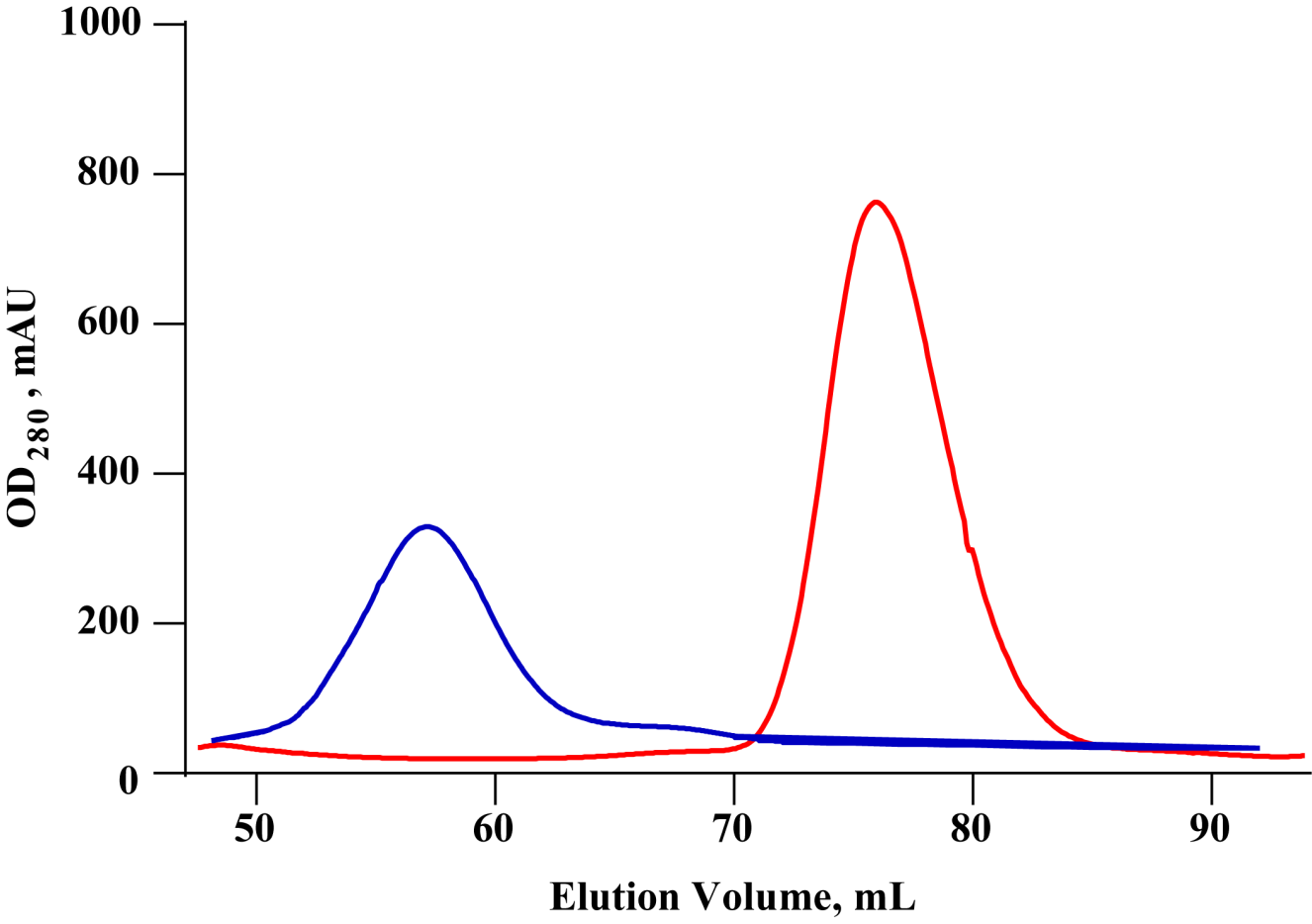


| Construct name | Elution Volume |
| --- | --- |
| TAT-MeCP2-eGFP | 57.1 mL |
| TAT-minMeCP2-eGFP | 75.9 mL |

**Supplementary Fig. 2** – GFC elution profiles of TAT-MeCP2-eGFP (marked in blue) and TAT-minMeCP2-eGFP (marked in red). The corresponding elution volumes are denoted in the inset table.


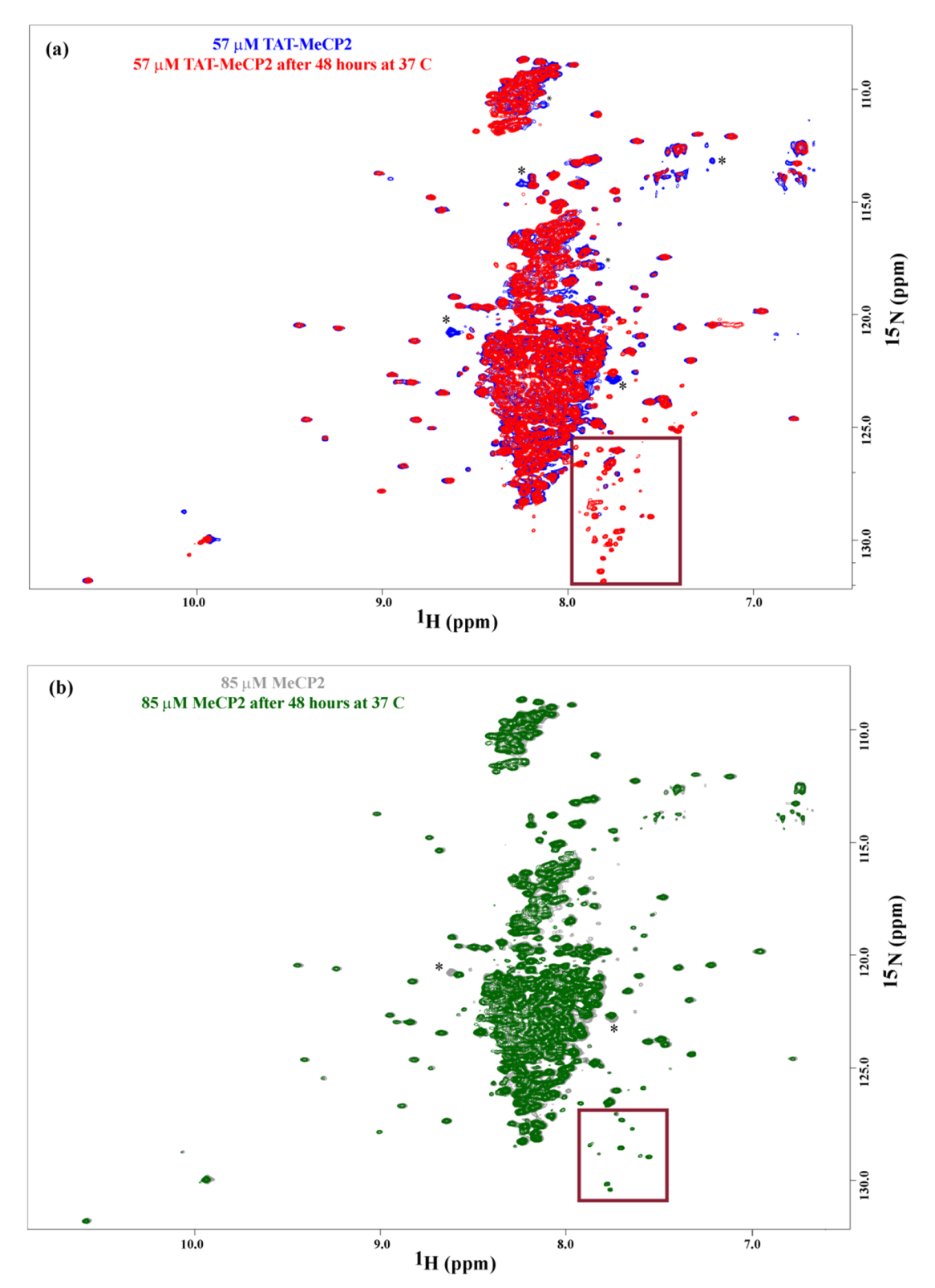


**Supplementary Fig. 3** – A 2D ^1^H, ^15^N TROSY-HSQC spectral overlays of **(A)** ^15^N labelled TAT-MeCP2 before (marked in blue) and after (marked in red) 72 hour incubation at 37 °C and **(B)** ^15^N labelled MeCP2 before (marked in grey) and after (marked in green) 72 hour incubation at 37 °C. Signals associated with protein degradation are boxed in both spectra. All spectra were acquired on a Bruker Avance 3 HD+ 800 MHz spectrometer at 25 °C.

**
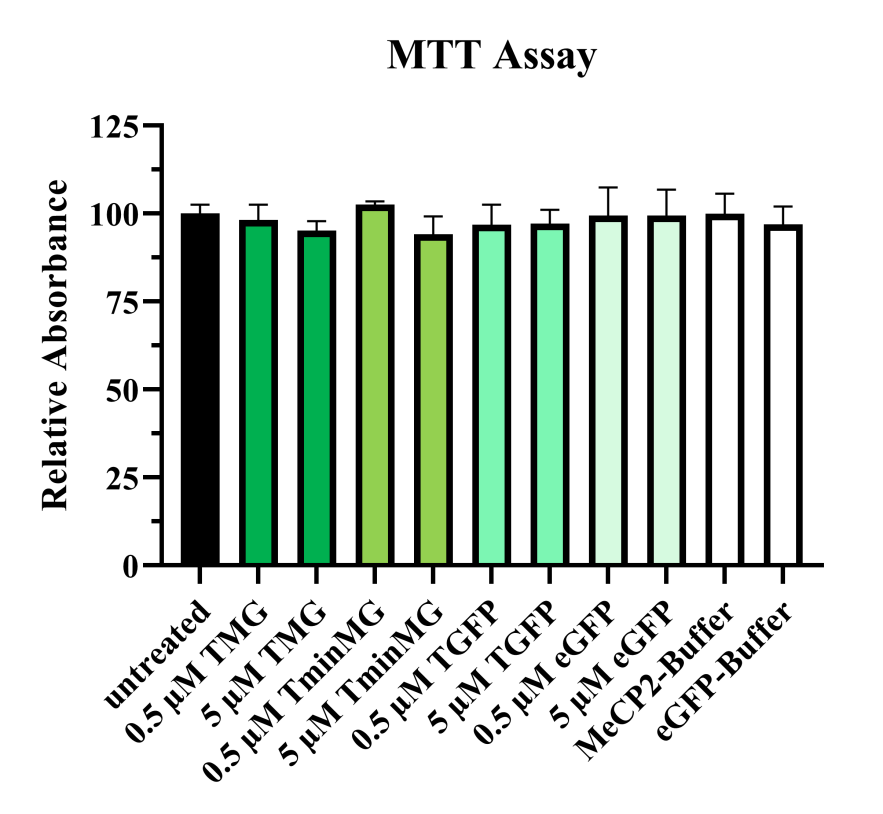
**

**Supplementary Fig. 4 –** Cell viability assessment using a MTT Assay. NIH3T3 cells were treated with 0.5 and 5 µM of TMG (TAT-MeCP2-eGFP), TminMG (TAT-minMeCP2-eGFP), TGFP (TAT-eGFP), eGFP as well as MeCP2 (DPBS, 200 mM NaCl, 10% (v/v) glycerol, 0.05% (w/v) CHAPS, pH = 7.2) and eGFP (DPBS, 10% (v/v) glycerol, pH = 7.2) protein storage buffers. Cell survival was determined using the MTT assay in triplicates. Conditions were normalized to untreated cells. No significant changes in viability were observed.


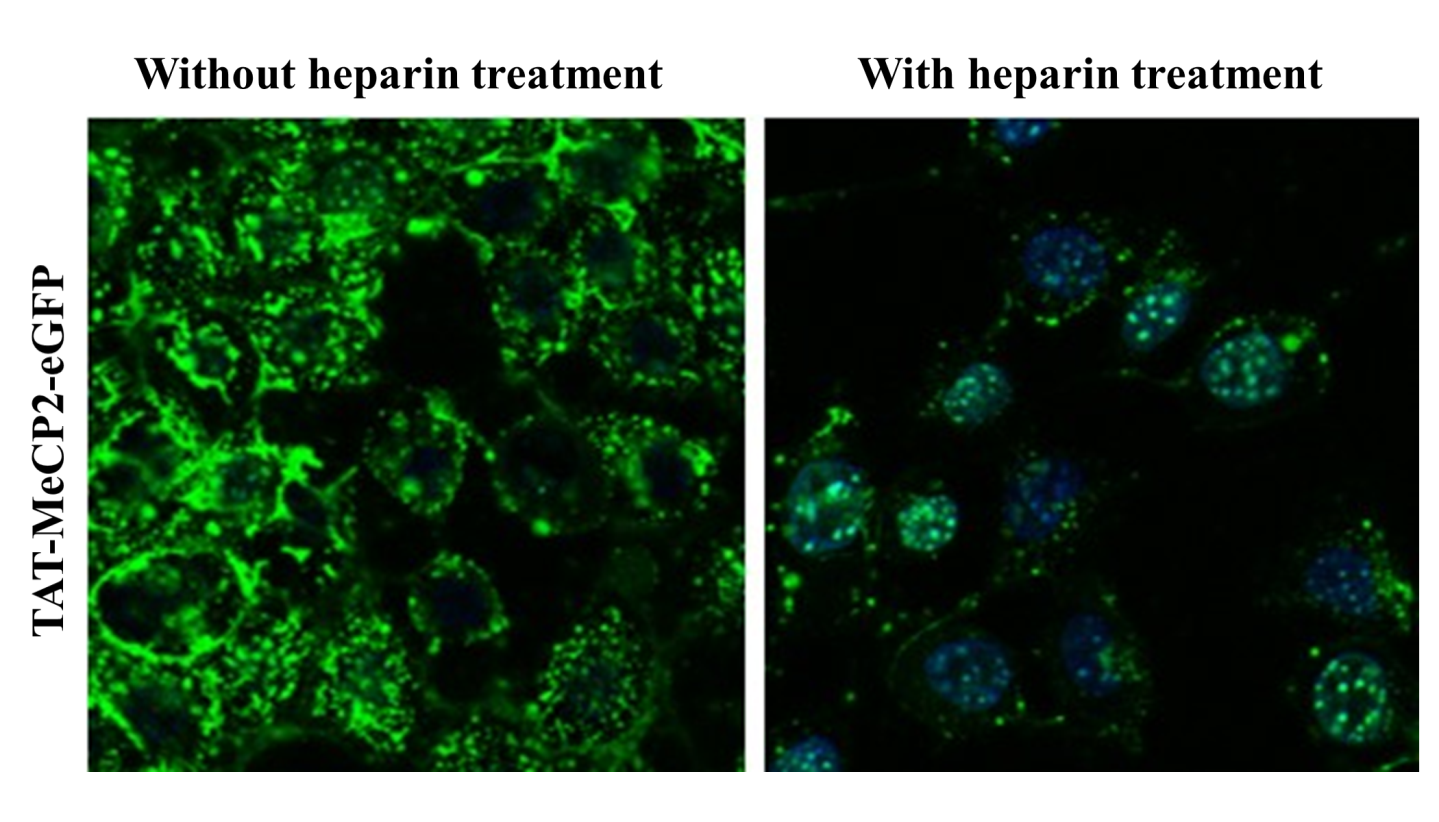


**Supplementary Fig. 5** – Effect of heparin treatment on external membrane bound TAT-MeCP2-eGFP. Living NIH3T3 cells were incubated with 5 μM of TAT-MeCP2-eGFP (green) for one hour and the nucleus was visualized with Hoechst 33342 (blue). Living cells were washed with DPBS and NIH3T3 were either immediately used for imaging (left panel) or subjected to three washes with 0.5 mg/mL heparin-DPBS (right panel). Confocal images show representative snapshots of living cells and a strong effect of heparin treatment on the removal of membrane-bound TAT-MeCP2-eGFP.

| Time Domain (F2, F1) | 2048/300 |
| --- | --- |
| Acquisition times F2/F1 (ms) | 79.9/52.9 |
| Sweep width F2/F1 (kHz) | 12.82/2.84 |
| Relaxation delay (sec) | 1.4 |
| Number of scans | 128 |
| Experiment time (hours) | 16.7 |

**Supplementary Table 1** – TROSY-HSQC acquisition parameters. Spectra were acquired on a Bruker Avance 3 HD+ 800 MHz spectrometer at 25 °C.

|  | 1 | 2 | 3 | 4 | 5 | 6 | 7 | 8 | 9 |
| --- | --- | --- | --- | --- | --- | --- | --- | --- | --- |
| A | **DPBS pH=7.2** | DPBS pH=7.2 | DPBS pH=7.2 | DPBS pH=7.2 | DPBS pH=7.2 | DPBS pH=7.2 | DPBS pH=7.2 | DPBS pH=7.2 | DPBS pH=7.2 |
|  | **10% Glycerol** | 10% Glycerol | 10% Glycerol | 10% Glycerol | 10% Glycerol | 10% Glycerol | 10% Glycerol | 10% Glycerol | 10% Glycerol |
|  | **200 mM NaCl** | 400 mM NaCl | 200 mM NaCl | 400 mM NaCl | 200 mM KCl | 400 mM NaCl | 200 mM KCl | 400 mM KCl | 400 mM NaCl |
|  | **0.05% CHAPS** | 0.05% CHAPS | 1 mM DTT | 1 mM DTT | 0.05% CHAPS | 0.05% CHAPS | 1 mM DTT | 1 mM DTT | 1 mM DTT |
|  |  |  | 0.05% CHAPS | 0.05% CHAPS |  |  | 0.05% CHAPS | 0.05% CHAPS | 0.05% PEG400 |
|  |  |  |  |  |  |  |  |  | 0.05% CHAPS |
| Intensity | **0.81** | 0.9 | 0.81 | 1.01 | 0.89 | 0.88 | 0.9 | 1.09 | 0.96 |
| Radius | **0.91** | 1.64 | 0.69 | 0.38 | 0.89 | 0.64 | 0.65 | 0.79 | 0.61 |
| B | DPBS pH=7.2 | DPBS pH=7.2 | DPBS pH=7.2 | DPBS pH=7.2 | DPBS pH=7.2 | DPBS pH=7.2 | DPBS pH=7.2 | DPBS pH=7.2 | DPBS pH=7.2 |
|  | 10% Glycerol | 10% Glycerol | 10% Glycerol | 10% Glycerol | 10% Glycerol | 10% Glycerol | 10% Glycerol | 10% Glycerol | 10% Glycerol |
|  | 200 mM NaCl | 400 mM NaCl | 200 mM NaCl | 400 mM NaCl | 200 mM KCl | 400 mM NaCl | 200 mM KCl | 400 mM KCl | 400 mM NaCl |
|  | 0.01% CYMAL®-5 | 0.01% CYMAL®-5 | 1 mM DTT | 1 mM DTT | 0.01% CYMAL®-5 | 0.01% CYMAL®-5 | 1 mM DTT | 1 mM DTT | 1 mM DTT |
|  |  |  | 0.01% CYMAL®-5 | 0.01% CYMAL®-5 |  |  | 0.01% CYMAL®-5 | 0.01% CYMAL®-5 | 0.05% PEG400 |
|  |  |  |  |  |  |  |  |  | 0.01% CYMAL®-5 |
| Intensity | 0.83 | 0.8 | 0.9 | 1 | 0.96 | 1.01 | 0.83 | 0.99 | 0.94 |
| Radius | 0.82 | 0.47 | 1.36 | 0.47 | 0.66 | 1.01 | 0.87 | 1.33 | 1.14 |
| C | DPBS pH=7.2 | DPBS pH=7.2 | DPBS pH=7.2 | DPBS pH=7.2 | DPBS pH=7.2 | DPBS pH=7.2 | DPBS pH=7.2 | DPBS pH=7.2 | DPBS pH=7.2 |
|  | 10% Glycerol | 10% Glycerol | 10% Glycerol | 10% Glycerol | 10% Glycerol | 10% Glycerol | 10% Glycerol | 10% Glycerol | 10% Glycerol |
|  | 200 mM NaCl | 400 mM NaCl | 200 mM NaCl | 400 mM NaCl | 200 mM KCl | 400 mM NaCl | 200 mM KCl | 400 mM KCl | 400 mM NaCl |
|  | 0.01% NTM | 0.01% NTM | 1 mM DTT | 1 mM DTT | 0.01% NTM | 0.01% NTM | 1 mM DTT | 1 mM DTT | 1 mM DTT |
|  |  |  | 0.01% NTM | 0.01% NTM |  |  | 0.01% NTM | 0.01% NTM | 0.05% PEG400 |
|  |  |  |  |  |  |  |  |  | 0.01% NTM |
| Intensity | 0.6 | 1 | 0.91 | 1 | 0.92 | 0.87 | 0.78 | 0.9 | 1.17 |
| Radius | 0.55 | 0.73 | 0.65 | 0.8 | 0.49 | 0.6 | 1.16 | 0.8 | 1.24 |
| D | DPBS pH=7.2 | DPBS pH=7.2 | DPBS pH=7.2 | DPBS pH=7.2 | DPBS pH=7.2 | DPBS pH=7.2 | DPBS pH=7.2 | DPBS pH=7.2 | DPBS pH=7.2 |
|  | 10% Glycerol | 10% Glycerol | 10% Glycerol | 10% Glycerol | 10% Glycerol | 10% Glycerol | 10% Glycerol | 10% Glycerol | 10% Glycerol |
|  | 200 mM NaCl | 400 mM NaCl | 200 mM NaCl | 400 mM NaCl | 200 mM KCl | 400 mM NaCl | 200 mM KCl | 400 mM KCl | 400 mM NaCl |
|  | 0.001% DDM | 0.001% DDM | 1 mM DTT | 1 mM DTT | 0.001% DDM | 0.001% DDM | 1 mM DTT | 1 mM DTT | 1 mM DTT |
|  |  |  | 0.001% DDM | 0.001% DDM |  |  | 0.001% DDM | 0.001% DDM | 0.05% PEG400 |
|  |  |  |  |  |  |  |  |  | 0.001% DDM |
| Intensity | 0.91 | 0.96 | 0.95 | 0.96 | 0.91 | 0.92 | 0.78 | 1 | 0.92 |
| Radius | 0.65 | 0.7 | 0.85 | 0.99 | 0.68 | 0.41 | 0.71 | 0.91 | 3.65 |

**Supplementary Table 2** – Buffer conditions tested for TAT-MeCP2-eGFP with intensity and radii ratios (before and after one week incubation at 25 °C) denoted. Values closer to 1 have a green background in the table heatmap. The selected buffer conditions (A1) for subsequent experiments are marked in bold. Percentages of glycerol and PEG 400 are given in v/v, detergent percentages are in w/v.
